# Supplementary material for: Validation of the Pain and Sensitivity Reactivity Scale in Neurotypical Late Adolescents and Adults
Source: Eur J Investig Health Psychol Educ. 2025 May 13;15(5):80. doi: 10.3390/ejihpe15050080 (PMC12109986; doi:10.3390/ejihpe15050080)
Supplement: Supplementary file 1 [file ejihpe-15-00080-s001.zip › ejihpe-3567028-supplementary.pdf]

**Supplementary Table S1**. Differences in the PSRS according to the gender.

| | Male | | Female | | | | |
|---|---|---|---|---|---|---|---|
| PSRS | M | (SD) | M | (SD) | t | p | d |
| Pain | 5.03 | (3.31) | 6.38 | (3.42) | -5.858 | .000** | -.40 |
| Total Sensory hyporeactivity | 14.49 | (8.64) | 12.80 | (9.00) | 2.792 | .002** | .19 |
| Hypo-Tactile | 4.43 | (2.84) | 4.33 | (3.12) | .487 | .313 | |
| Hypo-Olfactory | 2.85 | (2.04) | 2.94 | (2.20) | -5.577 | .282 | |
| Hypo-Visual | 2.29 | (2.02) | 1.86 | (2.07) | 3.095 | .001** | .21 |
| Hypo-Taste | 2.16 | (2.01) | 1.61 | (1.80) | 4.347 | .000** | .29 |
| Hypo-Auditory | 2.76 | (2.06) | 2.07 | (2.04) | 4.980 | .000** | .34 |
| Total Sensory hyperreactivity | 11.38 | (7.84) | 12.46 | (9.22) | -1.785 | .027* | -.13 |
| Hyper-Tactile | 2.66 | (2.34) | 3.09 | (2.69) | -2.448 | .007** | -.17 |
| Hyper-Olfactory | 2.55 | (2.05) | 2.93 | (2.48) | -2.387 | .008** | -.17 |
| Hyper-Visual | 1.38 | (1.60) | 1.27 | (1.63) | 1.048 | .147 | |
| Hyper-Taste | 1.68 | (1.81) | 1.91 | (2.31) | -1.544 | .042* | -.11 |
| Hyper- Auditory | 3.11 | (2.32) | 3.25 | (2.55) | -.857 | .195 | |
| Total PSRS | 30.90 | (16.27) | 31.64 | (18.12) | -.614 | .269 | |

Note. PSRS = Pain and Sensitivity Reactivity Scale; M= Mean; SD= Standard Deviation; Hypo= sensory hyporeactivity; Hyper= sensory hyperreactivity; t= Student's T; d= magnitude of the differences; p= statistical significance; * p < .05; ** p <.01.

**Supplementary Table S2.** Differences in the PSRS according to the age.

| | Late adolescents | | Adults | | | | |
|---|---|---|---|---|---|---|---|
| PSRS | M | (SD) | M | (SD) | t | p | d |
| Pain | 5.92 | (83.33) | 5.98 | (3.44) | -.294 | .384 | |
| Total Sensory hyporeactivity | 13.65 | (8.68) | 12.08 | (8.11) | 3.102 | .001** | .19 |
| Hypo-Tactile | 4.67 | (3.03) | 3.91 | (2.86) | 4.288 | .000** | .26 |
| Hypo-Olfactory | 2.98 | (2.15) | 2.71 | (2.04) | 2.128 | .017* | .13 |
| Hypo-Visual | 1.96 | (1.94) | 1.85 | (2.01) | .948 | .171 | |
| Hypo-Taste | 1.67 | (1.72) | 1.64 | (1.70) | .334 | .369 | |
| Hypo-Auditory | 2.38 | (2.04) | 1.97 | (1.83) | 3.485 | .000** | .21 |
| Total Sensory hyperreactivity | 12.35 | (8.25) | 11.39 | (8.53) | 1.893 | .030* | .11 |
| Hyper-Tactile | 3.08 | (2.48) | 2.70 | (2.42) | 2.569 | .005** | .16 |
| Hyper-Olfactory | 2.91 | (2.33) | 2.61 | (2.22) | 2.23 | .013* | .13 |
| Hyper-Visual | 1.26 | (1.47) | 1.25 | (1.53) | .168 | .433 | |
| Hyper-Taste | 2.00 | (2.14) | 1.62 | (2.09) | 2.914 | .002** | .18 |
| Hyper-Auditory | 3.10 | (2.31) | 3.21 | (2.54) | -.690 | .245 | |
| Total PSRS | 31.95 | (17.09) | 29.45 | (16.25) | 2.482 | .006** | .15 |

Note. PSRS = Pain and Sensitivity Reactivity Scale; M= Mean; SD= Standard Deviation; Hypo= sensory hyporeactivity; Hyper= sensory hyperreactivity; t= Student's T; d= magnitude of the differences; p= statistical significance; * p < .05; ** p <.01.
